# An explainable machine learning framework utilizing ultrasound radiomics for the preoperative differentiation between granulomatous lobular mastitis and breast cancer

**DOI:** 10.3389/fonc.2026.1641681

**Published:** 2026-04-24

**Authors:** Jinhong Zhou, Zhongcun Lai, Cishun Yu, Dan Liu, Yuguo Wei, Xiaowei Han, Guozheng Zhang

**Affiliations:** 1 Department of Ultrasound, The Quzhou Affiliated Hospital of Wenzhou Medical University (Quzhou People’s Hospital), Quzhou, China; 2 Department of Ultrasound, Zhejiang Medical & Health Group Quzhou Hospital (Zhejiang Quhua Hospital), Quzhou, China; 3 Department of Medical Affairs, Pharmaceutical Diagnosis, GE Healthcare, Hangzhou, China; 4 Department of Radiology, The Quzhou Affiliated Hospital of Wenzhou Medical University (Quzhou People’s Hospital), Quzhou, China

**Keywords:** breast cancer, granulomatous lobular mastitis, machine learning, mastitis, radiomics, SHAP

## Abstract

**Objective:**

To develop an interpretable machine learning model utilizing ultrasound radiomics for distinguishing between granulomatous lobular mastitis and breast cancer.

**Methods:**

This retrospective study encompassed 237 patients who underwent preoperative breast ultrasound examinations and received pathological diagnoses of either granulomatous lobular mastitis or breast cancer at Quzhou People’s Hospital between April 2013 and April 2023. Radiomic features were extracted from the ultrasound images, and feature selection was conducted using intra-class correlation coefficients, Pearson correlation coefficients, and the least absolute shrinkage and selection operator regression. Machine learning models based on radiomics were constructed using Extremely Randomized Trees, Light Gradient Boosting Machine, and Random Forest. Additionally, a combined model was developed by integrating independent clinical predictors with the radiomics signature.The model’s performance was assessed using the area under the receiver operating characteristic curve, accuracy, sensitivity, and specificity. To evaluate clinical utility, decision curve analysis was employed, while Shapley additive explanation was utilized to interpret model explainability.

**Results:**

A total of 1,161 radiomic features were extracted from each ultrasound image. Following Pearson correlation filtering, 135 features were retained, and 15 features were selected using the least absolute shrinkage and selection operator regression for model construction. The combined model, which integrated clinical factors with the radiomics signature, exhibited superior performance, achieving an AUC of 0.935 (95% *CI*: 0.902–0.969) in the training cohort and 0.833 (95% *CI*: 0.710–0.950) in the validation cohort. DCA indicated favorable clinical applicability. The Shapley additive explanation analysis shows that the imaging biomarker features lbp_3D_k_glszm_SmallAreaLowGrayLevelEmphasis, gradient_glcm_Imc2, gradient_glszm_ZoneEntropy, original_shape_Elongation, squareroot_glrlm_RunEntropy, and wavelet_HLH_glszm_LowGrayLevelZoneEmphasis have a strong correlation with the prediction of granulomatous lobular mastitis.

**Conclusion:**

The combined model, which incorporates ultrasound radiomics and clinical factors, exhibited significant efficacy in preoperatively differentiating granulomatous lobular mastitis from breast cancer. This non-invasive and interpretable methodology shows potential for enhancing diagnostic precision and guiding clinical decision-making.

## Introduction

Granulomatous lobular mastitis (GLM) is a benign inflammatory condition of the breast, distinguished by non-caseating lobular necrosis and granuloma formation, with an etiology that remains uncertain (1). The clinical presentation and imaging characteristics of GLM are varied (2). Ultrasound is the preferred initial imaging technique for diagnosing GLM. Nevertheless, the sonographic similarities between GLM and breast cancer frequently lead to misdiagnosis and subsequent delays in treatment (3).

Compared to ultrasound, mammography offers limited value in distinguishing breast cancer from GLM (4). Study indicates that approximately 55% of GLM cases are initially suspected to be breast cancer based on mammographic findings (5). Dynamic contrast-enhanced magnetic resonance imaging (DCE-MRI) serves as an adjunctive diagnostic modality, enhancing the differentiation between GLM and breast cancer when integrated with clinical assessments and conventional radiological evaluations (4, 6). Despite its utility, MRI is associated with high costs, specific contraindications, and the potential risk of allergic reactions to contrast agents. While pathological examination remains the definitive standard for diagnosis (7, 8), the procedure for obtaining tissue samples is invasive and may result in patient trauma. GLM and breast cancer require distinct therapeutic approaches. Breast cancer frequently requires extensive radical mastectomy, which substantially alters breast aesthetics. Conversely, GLM demands a tailored management strategy contingent upon clinical staging. Certain patients may exhibit favorable responses to pharmacological treatment alone, whereas others might require a combination of medication and surgical excision of lesions (9). Therefore, the ability to accurately and non-invasively differentiate GLM from breast cancer preoperatively is of significant clinical importance.

In recent years, artificial intelligence (AI) and deep learning techniques have been increasingly applied to breast imaging, demonstrating promising diagnostic performance across different imaging modalities. Comparative studies based on mammography have shown that convolutional neural network (CNN) architectures can achieve high accuracy in breast cancer classification, although performance varies depending on network design and feature representation (10, 11). In addition, AI-based mammography models have been reported to improve diagnostic efficiency while maintaining robust classification performance, highlighting their potential role as clinical decision-support tools (12).

Beyond mammography, deep learning approaches have also been successfully applied to ultrasound imaging for automated breast cancer detection, further supporting the feasibility of non-invasive, image-based diagnostic models (11). Meanwhile, machine learning frameworks integrating texture and intensity features have demonstrated improved classification accuracy in medical image analysis, underscoring the methodological foundation of radiomics-based approaches (13). However, variability in algorithm selection and model generalizability remains a challenge, as evidenced by comparative machine learning studies in other clinical domains (14).

Recent advances in hybrid deep learning models incorporating attention mechanisms have further enhanced feature representation and diagnostic accuracy by emphasizing task-relevant regions within medical images (15). These developments underscore the importance of combining advanced feature extraction with explainable modeling strategies to facilitate clinical translation.

Accordingly, this study aimed to develop predictive models to distinguish GLM from breast cancer by integrating ultrasound radiomics, multiple machine learning algorithms, and independent clinical risk factors. We constructed and evaluated a radiomics model, a clinical model, and a combined model. The combined model was presented as a nomogram, providing an intuitive and individualized tool for clinical decision-making. Furthermore, the study explored the interpretability of the machine learning radiomics model using Shapley Additive Explanations (SHAP), enabling transparent assessment of feature contributions at both global and individual levels.

## Materials and methods

### Ethical approval

This retrospective study received approval from the Ethics Committee of Quzhou People’s Hospital (Approval No.2025-005), with a waiver for the requirement of informed consent.

### Patients

**Figure 1:**
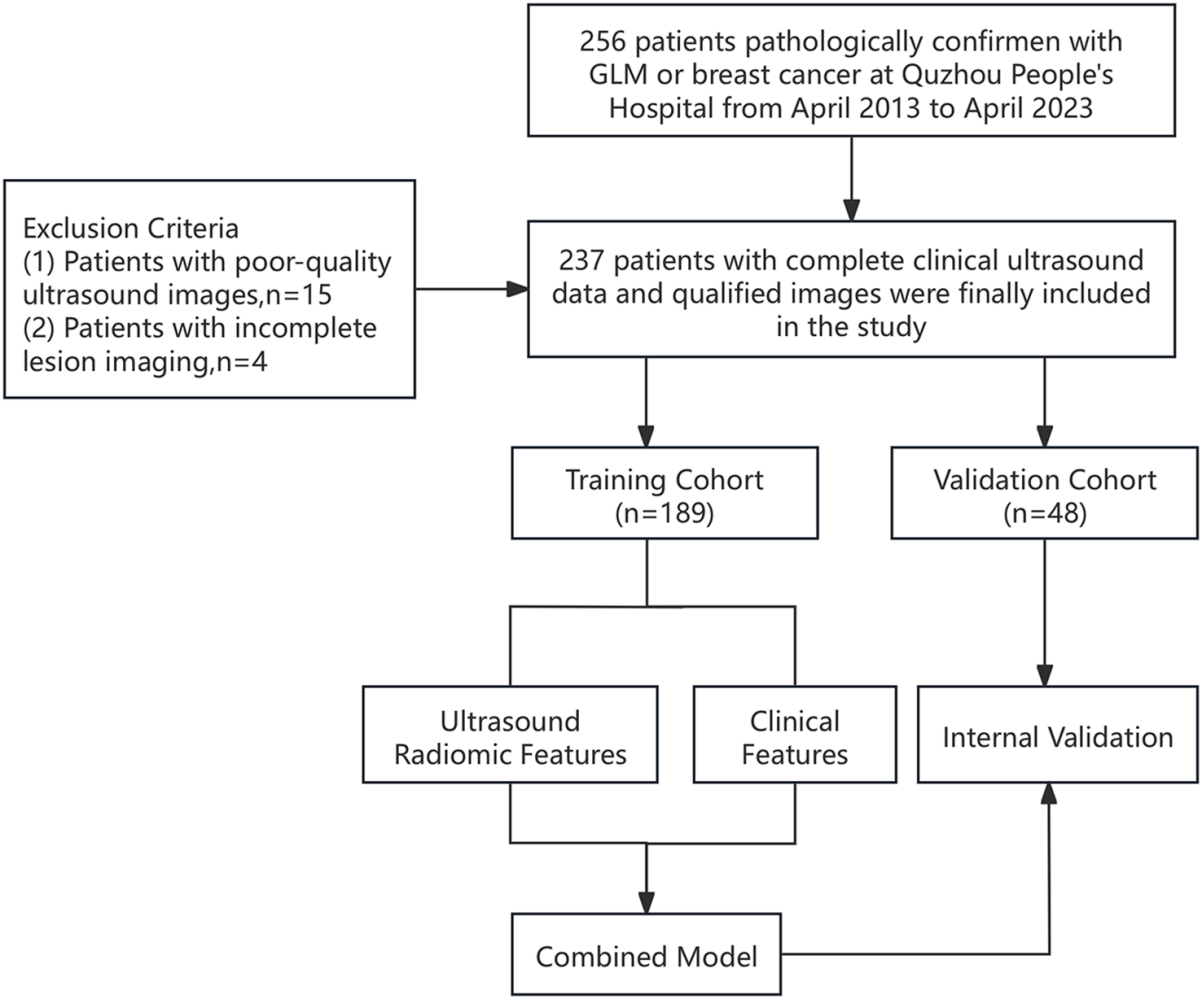
Study flowchart showing patient inclusion and exclusion.

A cohort of 237 patients, diagnosed with either GLM or breast cancer, was consecutively enrolled between April 2013 and April 2023. Each patient underwent breast ultrasound examinations and clinical data collection in the Department of Ultrasound, with diagnoses confirmed through pathological analysis. **Figure 1** illustrates the overall workflow of this study.

The inclusion criteria comprised the following: (1) a pathological confirmation of GLM or breast cancer obtained through core needle biopsy or surgical intervention; (2) a pre-treatment breast ultrasound examination; and (3) the availability of clear and analyzable ultrasound images. The exclusion criteria included: (1) ultrasound images of insufficient quality for analysis; (2) incomplete clinical data; and (3) lesions exceeding 6 cm in diameter that could not be fully visualized via ultrasound.

### Ultrasound image acquisition

Ultrasound examinations were conducted utilizing high-frequency transducers on the Philips IU22 and Philips EPIQ 5 systems (**Supplementary File S1**). Comprehensive scanning parameters are provided in **Supplementary Table 1**. All patients were examined in the supine position. The ultrasound images obtained were subsequently transferred to a workstation for further analysis. Two ultrasound doctors, with 3 years (LZC) and 15 years (ZJH) of experience in breast ultrasound, respectively, independently evaluated all lesions. In instances of disagreement, consensus was achieved through discussion. Both doctors were blinded to the pathological results.

### Feature selection, region of interest segmentation, and rad_signature construction

Clinical factor analysis, incorporating baseline clinical data and ultrasound signs, was conducted utilizing the independent samples t-test, Mann–Whitney U test, or chi-square (χ²) test, as deemed appropriate. Both univariate analysis and multivariate analyses were employed to compare clinical characteristics between the GLM and breast cancer cohorts. Clinical variables with a significance level of *p* < 0.05 were selected for inclusion in the development of the clinical model. Ultrasound images were stored in the Digital Imaging and Communications in Medicine (DICOM) format. A ultrasound doctor (L.Z.C.) manually delineated the region of interest (ROI) along the lesion boundary using ITK-SNAP software (version 3.6.0; www.itksnap.org). To evaluate inter-observer reproducibility, a second ultrasound doctor (Z.J.H.) re-annotated the ROIs on a randomly selected subset of 30 images following a two-week interval.

Radiomic features were extracted utilizing PyRadiomics (http://pyradiomics.readthedocs.io), an open-source Python package. The features extracted encompassed first-order statistics, shape features, gray-level co-occurrence matrix (GLCM), gray-level size zone matrix (GLSZM), gray-level run-length matrix (GLRLM), neighboring gray-tone difference matrix (NGTDM), and gray-level dependence matrix (GLDM). During the feature selection process, inter-observer reliability was initially assessed, and only those features exhibiting an intraclass correlation coefficient (ICC) exceeding 0.75 were retained to ensure reproducibility. Subsequently, the Pearson correlation coefficient was employed to evaluate the pairwise correlation among features; for any pair exhibiting a correlation coefficient greater than 0.9, one feature was retained to mitigate redundancy. Ultimately, the least absolute shrinkage and selection operator (LASSO) regression method was employed to construct the Rad_signature, with 10-fold cross-validation implemented to optimize the model.

### Model development and evaluation

Radiomics models were developed utilizing Extra Trees, Light Gradient Boosting Machine (LightGBM), and Random Forest (RF) classifiers. To mitigate the risk of overfitting, a five-fold cross-validation procedure was implemented within the training cohort to determine the optimal hyperparameters for each classification model. The final radiomic feature importance scores were extracted from the model demonstrating the highest performance. Independent clinical predictors, identified through multivariate analysis, were subsequently incorporated into the optimal classifier to construct the clinical predictive model. A combined model, integrating both clinical factors and the Rad_signature, was then established. To assess the predictive performance of these models, receiver operating characteristic (ROC) curves were generated for both the training and validation cohorts. Metrics such as the area under the ROC curve (AUC), accuracy, sensitivity, and specificity were computed. Furthermore, calibration curves and decision curve analysis (DCA) were utilized to evaluate the clinical utility of the models.

### Model visualization and interpretability

A nomogram that integrates ultrasound radiomics with the independent clinical predictors was developed to serve as a potential personalized tool for distinguishing GLM from breast cancer. To improve the transparency and clinical interpretability of the model’s decision-making process, SHAP were utilized to elucidate the trained machine learning model. SHAP, a game-theory-based methodology, quantifies the marginal contribution of each input variable to the model’s prediction. The fundamental concept of SHAP involves simulating feature permutations and calculating the weighted average contribution of each feature to a specific prediction, thereby ensuring additive and locally consistent explanations. In the SHAP summary plots, the final model prediction for each sample is decomposed into the sum of SHAP values associated with individual features. These values illustrate how the model derives a prediction by aggregating positive and negative contributions from various features relative to a baseline prediction.

This interpretive analysis facilitates the identification of key features, validates the logical framework of model decisions, and ultimately enhances the model’s credibility and applicability within clinical contexts.

### Statistical analysis

All statistical analyses were conducted using R software (version 3.6.0; http://www.r-project.org). Continuous variables were presented as mean ± standard deviation and analyzed using either the independent samples t-test or the Mann–Whitney–Wilcoxon test, contingent upon the data distribution. Categorical variables were assessed using the chi-square test or Fisher’s exact test, as deemed appropriate. The LASSO algorithm was applied via the “glmnet” package in R. ROC curves were generated using the “pROC” package, while DCA was executed with the “rmda” package. A two-sided p-value of less than 0.05 was considered to indicate statistical significance.

## Results

### Clinical and imaging characteristics

In this study, a total of 237 breast masses were examined, with extensive clinical and ultrasound imaging data collected. **Table 1** details the baseline characteristics of patients with GLM and breast cancer across both the training and validation cohorts. The training cohort consisted of 189 patients, including 105 with breast cancer and 84 with GLM, while the validation cohort comprised 48 patients, with 26 diagnosed with breast cancer and 22 with GLM.

**Table 1:** Patient baseline characteristics between training cohort and validation cohort.

Characteristics	Training cohort			Validation cohort		
	Breast cancer (n=105)	GLM (n=84)	*p*	Breast cancer (n=26)	GLM (n=22)	*p*
Age	42.10 ± 6.41	38.92 ± 6.76	<0.001	45.65 ± 5.97	40.55 ± 9.55	0.003
Nipple discharge	0.10 ± 0.29	0.05 ± 0.21	0.154	0.00 ± 0.00	0.05 ± 0.21	0.296
Longest diameter	2.13 ± 1.18	3.03 ± 1.20	<0.001	2.60 ± 2.06	2.64 ± 1.29	0.325
Shortest diameter	1.28 ± 0.65	1.56 ± 0.75	0.004	1.62 ± 1.06	1.46 ± 0.82	0.959
Inflammatory indicators			0.676			0.881
0	99.00 (94.29)	77.00 (91.67)		25.00 (96.15)	20.00 (90.91)	
1	6.00 (5.71)	7.00 (8.33)		1.00 (3.85)	2.00 (9.09)	
Breast pain			0.393			0.274
0	91.00 (86.67)	77.00 (91.67)		21.00 (80.77)	21.00 (95.45)	
1	14.00 (13.33)	7.00 (8.33)		5.00 (19.23)	1.00 (4.55)	
Skin lesions			1			0.178
0	98.00 (93.33)	78.00 (92.86)		26.00 (100.00)	19.00 (86.36)	
1	7.00 (6.67)	6.00 (7.14)		null	3.00 (13.64)	
Margin status			0.394			0.604
1	81.00 (77.14)	64.00 (76.19)		18.00 (69.23)	18.00 (81.82)	
2	5.00 (4.76)	9.00 (10.71)		2.00 (7.69)	1.00 (4.55)	
3	17.00 (16.19)	10.00 (11.90)		6.00 (23.08)	3.00 (13.64)	
4	2.00 (1.90)	1.00 (1.19)		null	null	
Angular status			0.142			0.092
0	96.00 (91.43)	70.00 (83.33)		19.00 (73.08)	21.00 (95.45)	
1	9.00 (8.57)	14.00 (16.67)		7.00 (26.92)	1.00 (4.55)	
Echogenic foci			0.750			1
0	41.00 (39.05)	30.00 (35.71)		13.00 (50.00)	11.00 (50.00)	
1	64.00 (60.95)	54.00 (64.29)		13.00 (50.00)	11.00 (50.00)	
Blood flow grade			0.512			0.700
0	1.00 (0.95)	1.00 (1.19)		null	1.00 (4.55)	
1	70.00 (66.67)	47.00 (55.95)		16.00 (61.54)	14.00 (63.64)	
2	12.00 (11.43)	12.00 (14.29)		5.00 (19.23)	3.00 (13.64)	
3	22.00 (20.95)	24.00 (28.57)		5.00 (19.23)	4.00 (18.18)	

Blood flow grade, the vascularity of the lesion and its periphery assessed by color Doppler ultrasound, graded as 0, no flow; 1, minimal flow (1–2 vessels); 2, moderate flow (3–4 vessels); 3, abundant flow (>4 vessels).

Age was found to have statistically significant clinical relevance in both univariate and multivariate analyses (p < 0.05). Conversely, other clinical features, such as nipple discharge, inflammatory markers, tenderness, transverse diameter of the nodule, echogenicity, margin characteristics, angular shape, and Doppler blood flow did not exhibit significant differences between the two groups (p > 0.05) within the clinical feature construction cohort. Ultimately, age emerged as the independent clinical risk factor, as shown in **Table 2**.

**Table 2:** Clinically significant factors and independent predictors.

Characteristics	Univariate logistic regression		Multiple-stepwise logistic regression analysis	
	OR (95%CI)	*p*	OR (95%CI)	*p*
Nipple discharge	-0.927 (-1.902;0.048)	0.118		
Breast pain	-0.693 (-1.902;0.048)	0.134		
Echogenic foci	-0.170 (-0.474;0.134)	0.358		
Skin lesions	-0.154 (-1.069;0.761)	0.782		
Margin status	-0.140 (-0.290;0.011)	0.127		
Blood flow grade	-0.054 (-0.186;0.077)	0.498		
Shortest diameter	-0.014 (-0.167;0.138)	0.878		
Age	-0.007 (-0.013;-0.001)	0.043	-0.007 (-0.013;-0.001)	0.043
Longest diameter	0.040 (-0.045;0.125)	0.436		
Inflammatory indicators	0.154 (-0.761;1.069)	0.782		
Angular status	0.442 (-0.261;1.145)	0.301		

### Radiomics feature selection and model construction

**Figure 2:**
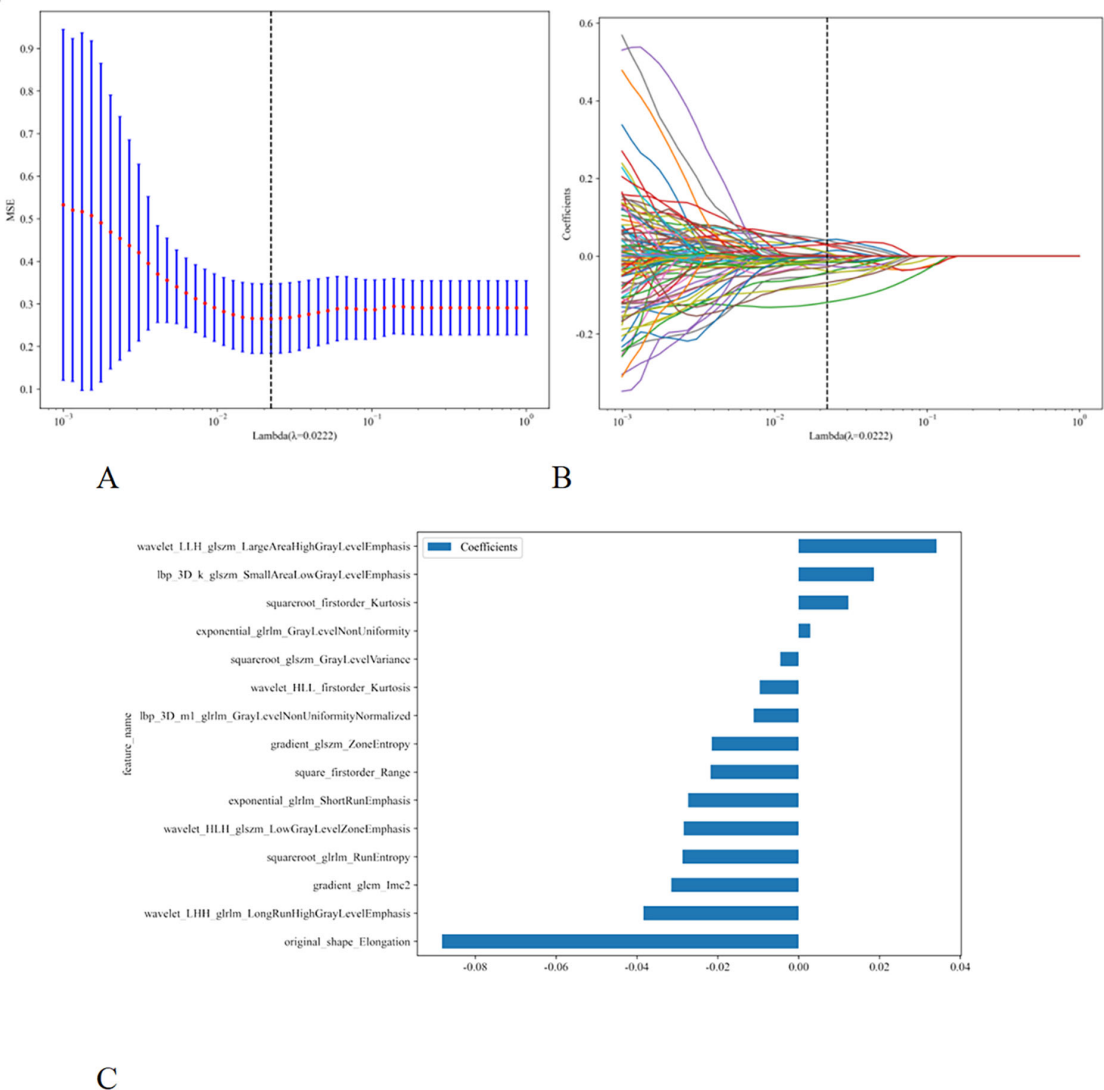
The figure illustrates the process of radiomics feature selection. **(A)** The feature selection of LASSO regression is based on the minimum binomial deviation of the model plus one standard error to review features with non-zero oefficients. **(B)** The 5-fold cross-validation process for LASSO regression feature selection is based on the minimum binomial deviation of the model plus one standard error, multiplied by the logarithm of l. This value is equal to 0.0222. **(C)** Proving the importance of specific radiomics features through histograms.

Initially, a total of 1,161 radiomic features were extracted from each ultrasound image. Following an assessment of inter-observer agreement using the ICC, 950 features with an ICC greater than 0.75 were retained. To further reduce redundancy, Pearson correlation analysis was conducted, resulting in the selection of 135 features. Dimensionality reduction was subsequently performed using LASSO, leading to the identification of the 15 most informative features and their corresponding coefficients for model construction (**Figure 2**). These selected features comprised 3 first-order features, 1 shape feature, and 11 high-order texture features. The values of these 15 features were incorporated into a formula to derive the Rad_signature, which reflects the total ablation value. The formula is presented as follows:

Rad_signature = 0.44725738396624476 +

+0.002853 * exponential_glrlm_GrayLevelNonUniformity

-0.027335 * exponential_glrlm_ShortRunEmphasis

-0.031500 * gradient_glcm_Imc2

-0.021501 * gradient_glszm_ZoneEntropy

+0.018597 * lbp_3D_k_glszm_SmallAreaLowGrayLevelEmphasis

-0.011159 * lbp_3D_m1_glrlm_GrayLevelNonUniformityNormalized

-0.088239 * original_shape_Elongation

-0.021780 * square_firstorder_Range

+0.012328 * squareroot_firstorder_Kurtosis

-0.028719 * squareroot_glrlm_RunEntropy

-0.004544 * squareroot_glszm_GrayLevelVariance

-0.028470 * wavelet_HLH_glszm_LowGrayLevelZoneEmphasis

-0.009643 * wavelet_HLL_firstorder_Kurtosis

-0.038413 * wavelet_LHH_glrlm_LongRunHighGrayLevelEmphasis

+0.034113 * wavelet_LLH_glszm_LargeAreaHighGrayLevelEmphasis

In this study, three distinct machine learning algorithms—Extra Trees, LightGBM, and RF—were employed to construct predictive models (**Table 3**). These algorithms were chosen due to their proven efficacy in managing complex datasets and delivering highly accurate predictions.

**Table 3:** Diagnostic performance of the radiomics based ML models in the training cohort and validation cohort.

Model	Accuracy	AUC (95% CI)	Sensitivity	Specificity	PPV	NPV
Training cohort						
Randomforest	0.836	0.912 (0.873-0.950)	0.857	0.819	0.791	0.878
Extratrees	0.767	0.856 (0.804-0.908)	0.857	0.695	0.692	0.859
LightGBM	0.878	0.930 (0.895-0.966)	0.893	0.867	0.843	0.910
Validation cohort						
Randomforest	0.750	0.787 (0.656-0.918)	0.818	0.692	0.692	0.818
Extratrees	0.688	0.736 (0.595-0.877)	0.636	0.731	0.667	0.704
LightGBM	0.708	0.810 (0.689-0.932)	0.818	0.615	0.643	0.800

**Figure 3:**
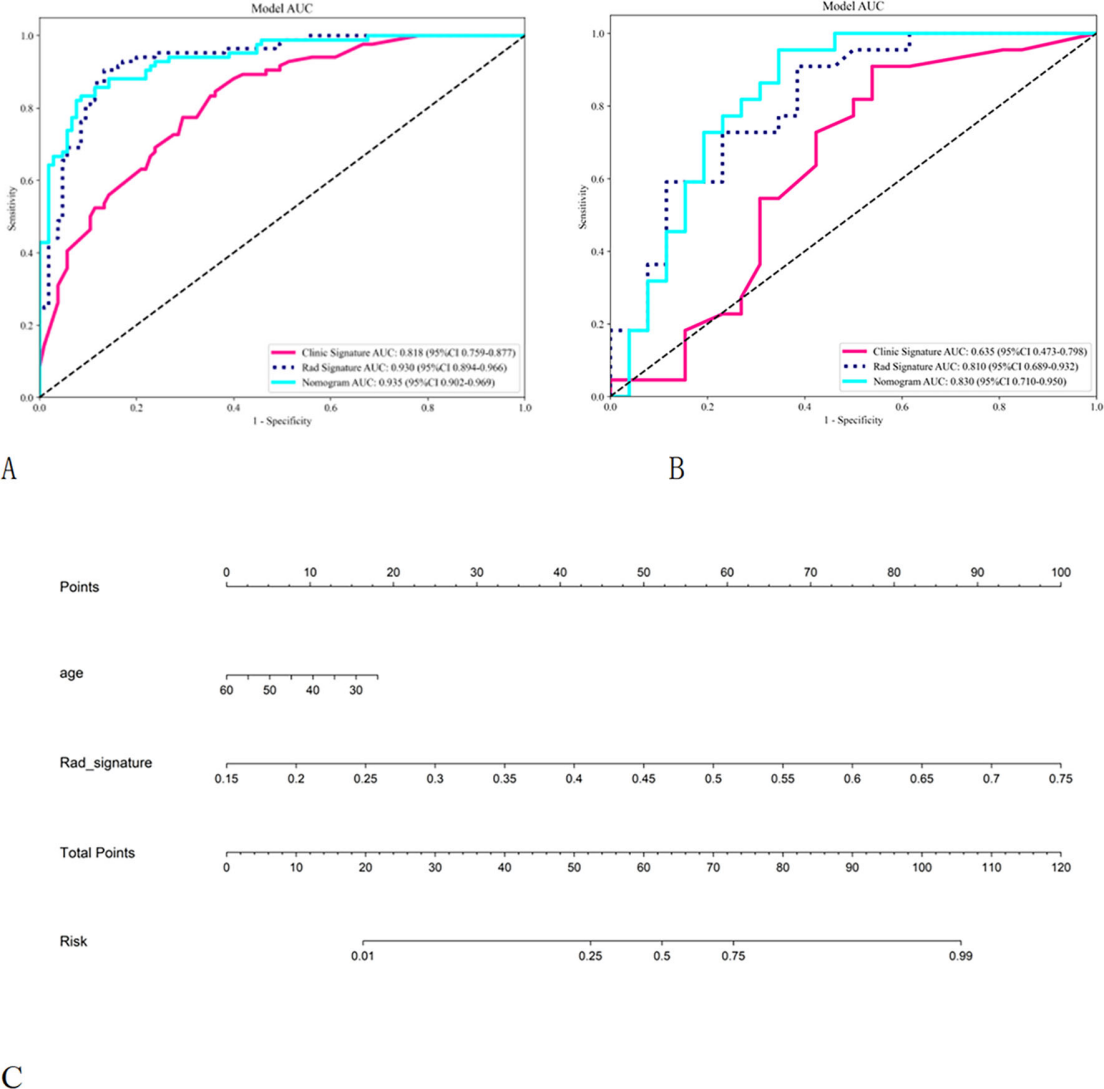
ROC curves of the training and validation cohorts for clinical models, Radomics models, and combined models, Nomogram based on a combined model for differentiating granulomatous lobular mastitis from breast cancer.

Among the models evaluated, the radiomics model developed utilizing the LightGBM algorithm exhibited superior predictive performance in both the training and validation cohorts, achieving an area under the curve (AUC) of 0.93 (95% [CI]: 0.895–0.966) and 0.81 (95% CI: 0.689–0.932), respectively. As a result, LightGBM was selected for the construction of the subsequent clinical model and the combined model. **Table 4** presents a comparative analysis of the predictive performance of the clinical model, radiomics model, and combined model, all developed using the LightGBM algorithm. ROC curve analysis revealed that the combined model attained an AUC of 0.935 (95% CI: 0.902–0.967) in the training cohort, indicating excellent discriminative capability (**Figure 3**). Within the training cohort, the combined model’s sensitivity, specificity, positive predictive value (PPV), and negative predictive value (NPV) for predicting mastitis were 82.1%, 91.4%, 88.5%, and 86.5%, respectively. In the validation cohort, these metrics were 90.9%, 65.4%, 69.0%, and 89.5%, respectively.

**Table 4:** The model predicts the effectiveness of GLM.

Model	AUC (95% CI)	Accuracy	Sensitivity	Specificity	PPV	NPV
Training cohort						
Clinical model	0.818 (0.759-0.877)	0.725	0.833	0.638	0.648	0.827
Radiomics model	0.930 (0.895-0.966)	0.878	0.893	0.867	0.843	0.910
Combined model	0.935 (0.902-0.969)	0.873	0.821	0.914	0.885	0.865
Validation cohort						
Clinical model	0.635 (0.473-0.798)	0.625	0.818	0.462	0.562	0.750
Radiomics model	0.810 (0.689-0.932)	0.708	0.818	0.615	0.643	0.800
Combined model	0.830 (0.710-0.950)	0.771	0.909	0.654	0.690	0.895

Across both the training and validation cohorts, the combined model demonstrated consistently superior predictive performance compared to the clinical model and the radiomics model. The DeLong test further validated that the differences in AUC values were statistically significant (**Supplementary File S2**).The DCA curve and calibration curve demonstrate significant clinical application value (**Supplementary File S3**).

### Model interpretability

**Figure 4:**
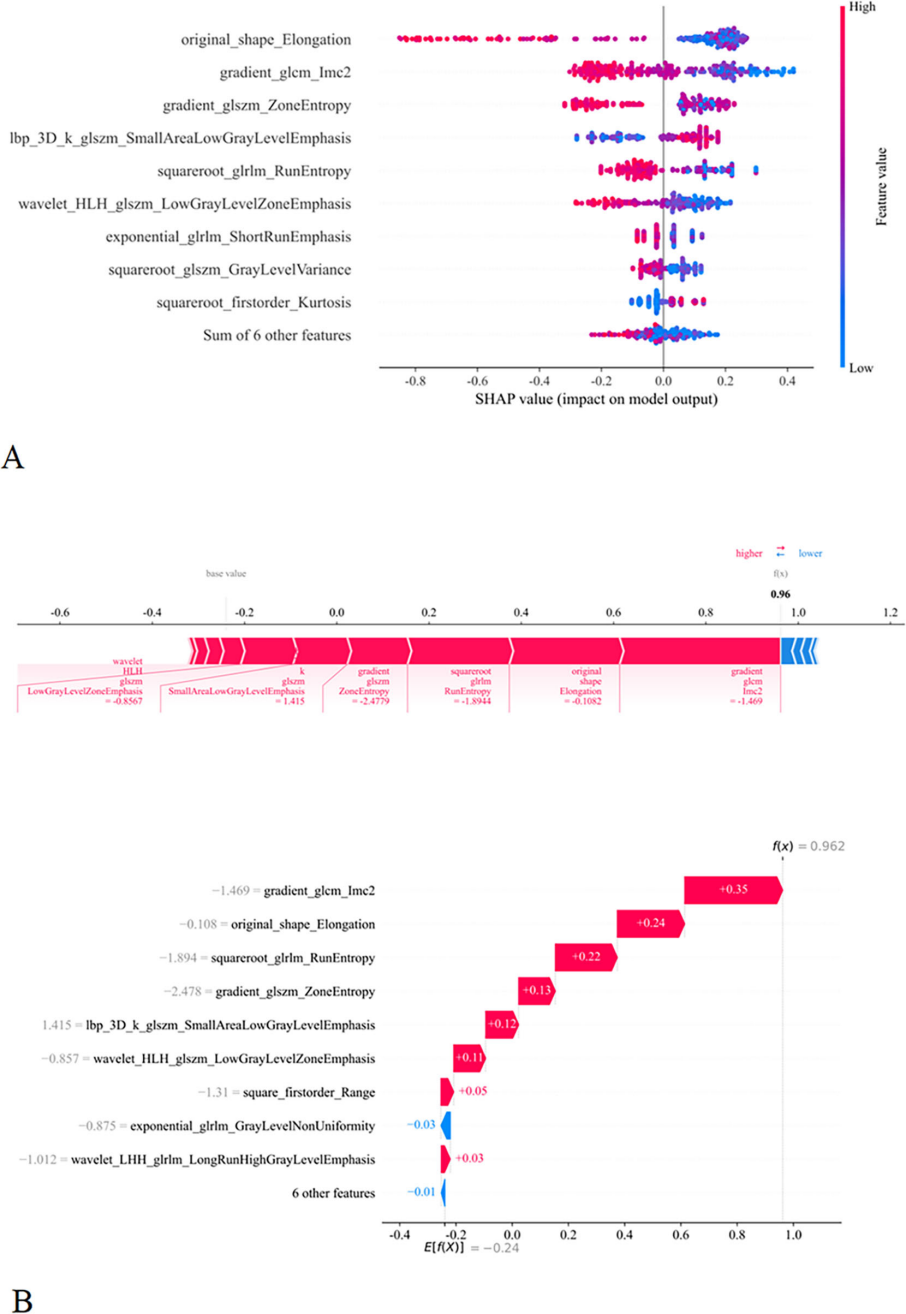
SHAP summary plot. **(A)** Ranking the importance of each feature in the final model output. **(B)** SHAP values of each feature in the final model. The different colors (red and blue) represent different levels of effect on the output of the model.

To further elucidate the mechanisms by which radiomic features contribute to model predictions, SHAP was utilized to interpret the classification model. **Figure 4A** displays the SHAP force plot for a sample from the generalized linear model (GLM), which visually dissects the predicted value at the individual level and illustrates both the global significance of features and the relationship between feature values and their corresponding SHAP values. **Figure 4B** presents the SHAP decision plot for the same sample, mapping the progression of the prediction value from the baseline to the final output along a decision path. The SHAP analysis indicated that the model’s high-risk predictions were predominantly influenced by image texture and shape features. Key contributing features included lbp_3D_k_glszm_SmallAreaLowGrayLevelEmphasis, gradient_glcm_Imc2, gradient_glszm_ZoneEntropy, original_shape_Elongation, squareroot_glrlm_RunEntropy, and wavelet_HLH_glszm_LowGrayLevelZoneEmphasis. Among these, complex texture features, such as those derived from the gray-level co-occurrence matrix (GLCM), and structural descriptors like elongation, played a pivotal role in the model’s decision-making process.

## Discussion

In this retrospective study, we developed and validated a noninvasive, individualized model based on ultrasound radiomics for differentiating GLM from breast cancer. The proposed model demonstrated favorable diagnostic performance, achieving an AUC of 0.935 in the training cohort and 0.833 in the validation cohort. Although a decline in performance was observed in the validation cohort, the model maintained acceptable discriminative ability, indicating its potential clinical value. To further enhance transparency and clinical interpretability, SHAP was applied to elucidate the contribution of individual radiomic features to model predictions. The final combined model was presented as a nomogram, providing clinicians with an intuitive and personalized decision-support tool.

Ultrasound is widely used as a first-line imaging modality for the screening and initial evaluation of breast diseases because of its accessibility, cost-effectiveness, and real-time imaging capability. GLM commonly manifests as an irregular hypoechoic mass with tubular extensions or confluent areas, reflecting its tendency to involve lobular units rather than exhibit destructive infiltrative growth (16–20). However, these sonographic features substantially overlap with those of breast cancer, including irregular shape, ill-defined margins, and increased vascularity, which frequently leads to misdiagnosis (21–25). Given that the diagnostic accuracy of fine-needle aspiration cytology may be as low as 21% (26), histopathological examination remains the gold standard. In cases where core needle biopsy is inconclusive, excisional biopsy is often required (27). Therefore, the development of an accurate and noninvasive method for early differentiation between GLM and breast cancer is of considerable clinical importance.

Radiomics has shown promise in capturing high-dimensional quantitative features that reflect tissue heterogeneity and microstructural characteristics beyond visual assessment, thereby improving differentiation between benign and malignant breast lesions (7, 27, 28). Previous ultrasound-based radiomics studies have reported diagnostic accuracies ranging from 76.3% to 79.8% (28) and approximately 79.9% (7) in distinguishing inflammatory breast conditions from malignancy. However, many traditional machine-learning models function as “black boxes,” which hampers their clinical acceptance (29, 30). To address this limitation, SHAP—an explainability framework grounded in cooperative game theory—was employed to provide both global and individual-level interpretability of the LightGBM model (31–33). SHAP has been successfully applied in various clinical scenarios, including the prediction of intraoperative hypoxemia (34) and COVID-19 outcomes (35). In line with recent advances in explainable artificial intelligence, attention-based deep learning architectures have been shown to improve model transparency and reliability, such as pyramidal attention networks for histopathological image analysis (36).

In this study, age was reaffirmed as an important clinical variable for differentiating GLM from breast cancer (37), consistent with prior evidence that GLM predominantly affects younger women, whereas breast cancer is more prevalent among middle-aged and older populations. In contrast, conventional ultrasound features such as margin, vascularity, and angular characteristics showed limited discriminative value when considered independently. These findings highlight the necessity of integrating clinical factors with radiomic features. SHAP-based global analysis indicated that model predictions were largely driven by higher-order texture features reflecting grayscale heterogeneity, textural complexity, and structural organization, which are known to differ between inflammatory and malignant breast lesions.

At the individual level, texture features such as gradient_glcm_Imc2, glszm_ZoneEntropy, and glrlm_RunEntropy exhibited positive contributions toward GLM prediction, suggesting relatively orderly gray-level distributions and lower intralesional complexity—hallmarks of chronic inflammatory processes. Morphological features such as original_shape_Elongation also contributed positively, indicating more regular lesion contours. In contrast, features including square_firstorder_Range, GrayLevel_NonUniformity, and LongRunHighGrayLevelEmphasis contributed negatively and were associated with malignancy-related characteristics such as internal heterogeneity, necrosis, or complex tissue architecture.

Notably, three different machine learning algorithms were employed for radiomics model construction in this study, among which the Light Gradient Boosting Machine demonstrated the best and most stable predictive performance. Accordingly, SHAP-based interpretability analysis was performed at both the global and individual levels for this model. The explainability provided by SHAP offers transparent, feature-level insights into model decision-making, thereby enhancing clinicians’ understanding of and confidence in the model’s predictions. Recent studies have further demonstrated that integrating optimization strategies with deep learning models, such as electromagnetic interaction-based feature selection combined with adaptive kernel attention networks, can significantly improve classification performance in high-dimensional datasets (38). Similarly, hybrid deep learning models incorporating attention mechanisms and statistical validation have shown enhanced segmentation accuracy in ultrasound imaging tasks (39).

Overall, the proposed radiomics–clinical combined model shows promising discriminative capability for the preoperative differentiation of granulomatous lobular mastitis from breast cancer. In the broader context of biomedical artificial intelligence, recent frameworks such as CICADA (UCX) integrate tumor segmentation, classification, and aggressiveness assessment into a unified architecture, demonstrating the potential of multi-task learning for improving diagnostic performance and interpretability (40). Moreover, comprehensive reviews on machine learning and deep learning models for anti-cancer drug response prediction emphasize the importance of robust validation and multimodal data integration for clinical translation (41). Future studies should focus on larger, multicenter datasets and external validation to further improve the robustness and clinical applicability of the model.

### Limitations

This study has several limitations. First, as a retrospective single-center study with internal validation only, the generalizability of the proposed model is limited; future multicenter studies with independent external validation are planned to enhance its applicability. Second, although the model demonstrated good performance in the training cohort, a decrease in performance was observed in the validation cohort, suggesting a potential risk of overfitting in the context of a relatively limited sample size; this issue may be addressed by expanding the cohort in future studies. Finally, only solid lesions with clearly defined peritumoral regions were included and manual segmentation was used; future work will incorporate automated segmentation techniques to reduce manual workload and improve reproducibility.

## Conclusion

The combined model, which incorporates both Rad_signature and clinical features, enhances the preoperative differentiation between GLM and breast cancer. In comparison to models that depend exclusively on ultrasound radiomics or clinical features, this combined approach exhibits superior predictive performance and holds potential as a valuable tool for supporting clinical decision-making.

## Data Availability

The data analyzed in this study is subject to the following licenses/restrictions: Patient Information Privacy Protection. Requests to access these datasets should be directed to GZ, zgz_0007@163.com.
