# Supplementary material for: An explainable machine learning framework utilizing ultrasound radiomics for the preoperative differentiation between granulomatous lobular mastitis and breast cancer
Source: Front Oncol. 2026 Apr 24;16:1641681. doi: 10.3389/fonc.2026.1641681 (PMC13152765; doi:10.3389/fonc.2026.1641681)

**Supplementary :**

**S1 | Equipment parameter**

| Equipment | Ultrasonic probe  (High-frequency linear array probe) | frequency range |
| --- | --- | --- |
| Philips IU22 Color Doppler Ultrasound equipped | L12-5 | 5–12 MHz |
| Philips EPIQ5 Color Doppler Ultrasound equipped | L18-4 | 4–18 MHz |

**S2 | Delong's test**

| Nomogram Vs Clinic | Nomogram Vs Rad |
| --- | --- |
| 8.863e-07 | 0.620 |
| 6.823e-03 | 0.603 |

Delong's Validation showed that the combined model had significant differences compared to both the clinical model and the omics model.

**
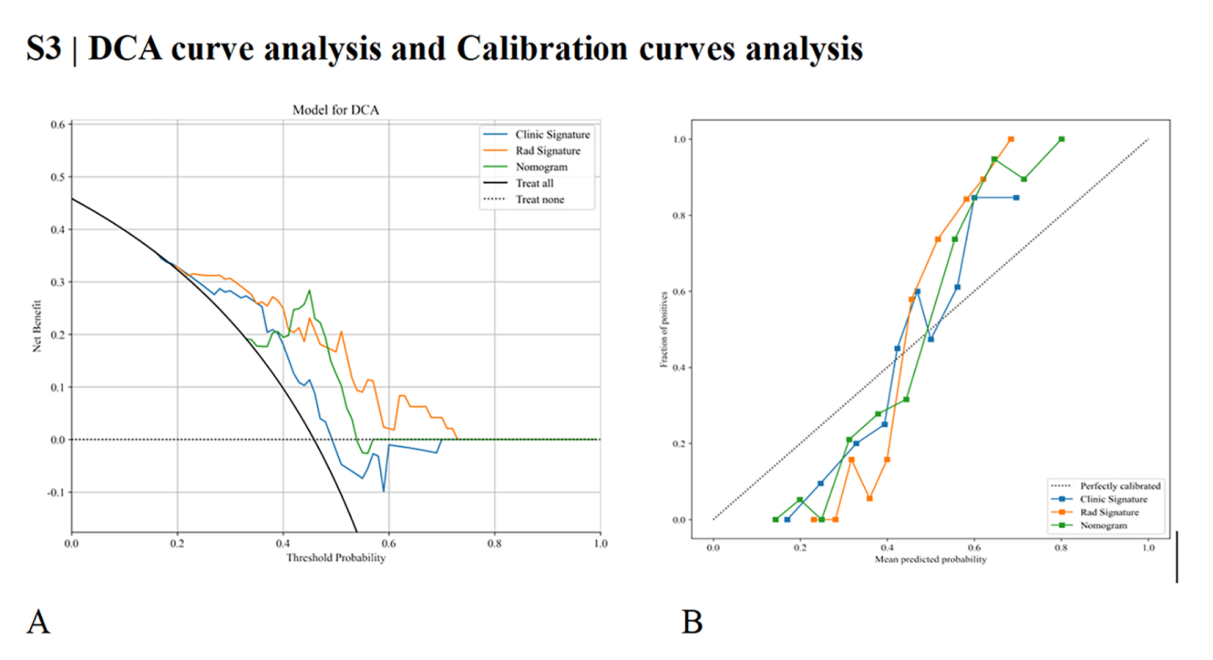
**

1. Decision curve analyses for the radiomics model, clinical-semantic model and combined model in the training cohort.(B)Calibration curves of the nomogram in the training cohorts.

**S4 | 5-fold cross-validation (CV) on the training cohort**

| **Model** | **Fold** | **Accuracy** | **AUC** | **Sensitivity** | **Specificity** | **PPV** | **NPV** |
| --- | --- | --- | --- | --- | --- | --- | --- |
| Rad_RF | 1 | 0.789473684 | 0.901960784 | 0.705882353 | 0.857142857 | 0.8 | 0.782608696 |
| Rad_RF | 2 | 0.842105263 | 0.918767507 | 0.705882353 | 0.952380952 | 0.923076923 | 0.8 |
| Rad_RF | 3 | 0.710526316 | 0.865546218 | 0.647058824 | 0.761904762 | 0.6875 | 0.727272727 |
| Rad_RF | 4 | 0.789473684 | 0.904761905 | 0.705882353 | 0.857142857 | 0.8 | 0.782608696 |
| Rad_RF | 5 | 0.835256024 | 0.912457206 | 0.857423501 | 0.819360124 | 0.791256324 | 0.878245325 |
| Clinic_LGBM | 1 | 0.763157895 | 0.809131652661064 | 0.588235294 | 0.904761905 | 0.833333333 | 0.730769231 |
| Clinic_LGBM | 2 | 0.710526316 | 0.813137254901961 | 0.588235294 | 0.80952381 | 0.714285714 | 0.708333333 |
| Clinic_LGBM | 3 | 0.725263142 | 0.818465365 | 0.833023592 | 0.638156892 | 0.648368246 | 0.827102385 |
| Clinic_LGBM | 4 | 0.684210526 | 0.742296919 | 0.470588235 | 0.857142857 | 0.727272727 | 0.666666667 |
| Clinic_LGBM | 5 | 0.648648649 | 0.75 | 0.5 | 0.761904762 | 0.615384615 | 0.666666667 |
| Nomo | 1 | 0.842105263 | 0.913165266 | 0.823529412 | 0.857142857 | 0.823529412 | 0.857142857 |
| Nomo | 2 | 0.873205348 | 0.935463512 | 0.821125681 | 0.914389021 | 0.885450236 | 0.865156802 |
| Nomo | 3 | 0.921052632 | 0.926778711484594 | 0.941176471 | 0.904761905 | 0.888888889 | 0.95 |
| Nomo | 4 | 0.842105263 | 0.899159664 | 0.764705882 | 0.904761905 | 0.866666667 | 0.826086957 |
| Nomo | 5 | 0.891891892 | 0.9175 | 0.8125 | 0.952380952 | 0.928571429 | 0.869565217 |
| Rad_ET | 1 | 0.684210526 | 0.855142857142857 | 0.352941176470588 | 0.952380952 | 0.857142857 | 0.64516129 |
| Rad_ET | 2 | 0.657894737 | 0.831932773 | 0.352941176 | 0.904761905 | 0.75 | 0.633333333 |
| Rad_ET | 3 | 0.710526316 | 0.764705882 | 0.411764706 | 0.952380952 | 0.875 | 0.666666667 |
| Rad_ET | 4 | 0.736842105 | 0.868347339 | 0.470588235 | 0.952380952 | 0.888888889 | 0.689655172 |
| Rad_ET | 5 | 0.767231024 | 0.856132524 | 0.857124563 | 0.695253652 | 0.692123524 | 0.859256324 |
| Rad_LGBM | 1 | 0.789473684 | 0.918767507 | 0.588235294 | 0.952380952 | 0.909090909 | 0.740740741 |
| Rad_LGBM | 2 | 0.815789474 | 0.924369748 | 0.647058824 | 0.952380952 | 0.916666667 | 0.769230769 |
| Rad_LGBM | 3 | 0.868421053 | 0.918767507 | 0.882352941 | 0.857142857 | 0.833333333 | 0.9 |
| Rad_LGBM | 4 | 0.815789474 | 0.915966387 | 0.764705882 | 0.857142857 | 0.8125 | 0.818181818 |
| Rad_LGBM | 5 | 0.878126532 | 0.932463542 | 0.893236541 | 0.867265312 | 0.843123521 | 0.912863501 |


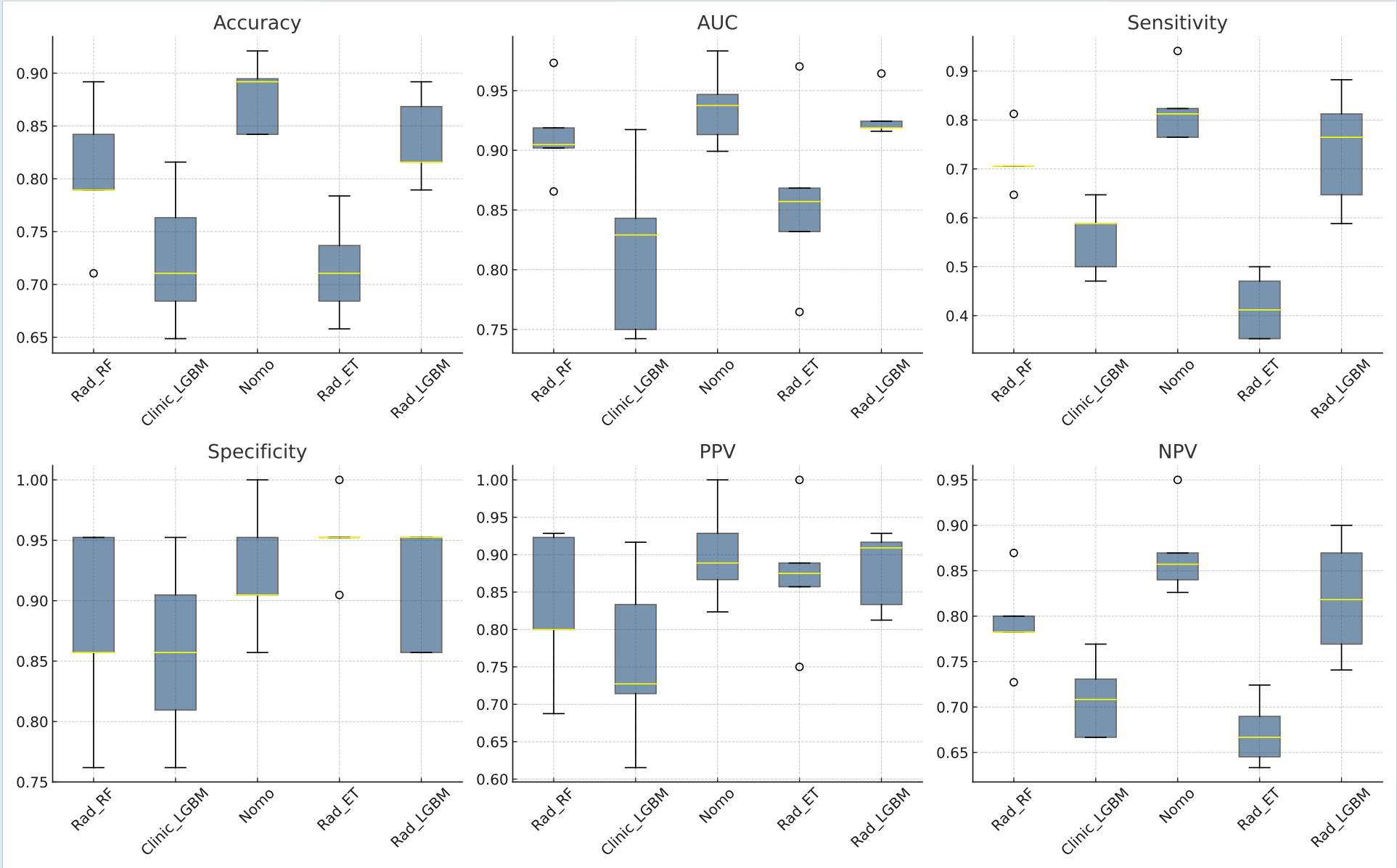

Supplement: Supplementary file 1 [file DataSheet1.docx]
