# Supplementary material for: An explainable machine learning framework utilizing ultrasound radiomics for the preoperative differentiation between granulomatous lobular mastitis and breast cancer
Source: Front Oncol. 2026 Apr 24;16:1641681. doi: 10.3389/fonc.2026.1641681 (PMC13152765; doi:10.3389/fonc.2026.1641681)
Supplement: Supplementary file 2 [file DataSheet2.docx]

Supplementary Data Analysis Report

GLM vs Breast Cancer Ultrasound Radiomics Study — Prepared in response to Reviewer 8 comments

# Section 1: Dataset Overview — Class Balance

The training set contains 105 Breast Cancer (BC) and 84 GLM cases; the test set contains 26 BC and 22 GLM cases. The BC:GLM ratio is 1.25:1 (training) and 1.18:1 (test), indicating mild class imbalance that does not warrant SMOTE or other augmentation.

**Table S0. Class distribution across train/test sets**

| **Set** | **BC (label=0)** | **GLM (label=1)** | **Total** | **BC:GLM** |
| --- | --- | --- | --- | --- |
| Train | 105 | 84 | 189 | 1.25:1 |
| Test | 26 | 22 | 48 | 1.18:1 |

# Section 2: Complete Performance Metrics (Table S1)

All five models were evaluated on both training and test sets. Metrics include AUC, accuracy (Acc), sensitivity (Sen), specificity (Spe), positive predictive value (PPV), negative predictive value (NPV), and F1-score. Note: Sen and Spe are computed with GLM (label=1) as the positive class.

**Table S1. Performance metrics for all models**

| **Model** | **Set** | **Acc** | **AUC** | **Sen** | **Spe** | **PPV** | **NPV** | **F1** |
| --- | --- | --- | --- | --- | --- | --- | --- | --- |
| Combined | Train | 0.873 | 0.935 | 0.821 | 0.914 | 0.885 | 0.865 | 0.852 |
| Combined | Test | 0.771 | 0.830 | 0.909 | 0.654 | 0.690 | 0.895 | 0.784 |
| Rad-RandomForest | Train | 0.836 | 0.912 | 0.857 | 0.819 | 0.791 | 0.878 | 0.823 |
| Rad-RandomForest | Test | 0.750 | 0.787 | 0.818 | 0.692 | 0.692 | 0.818 | 0.750 |
| Rad-ExtraTrees | Train | 0.767 | 0.856 | 0.857 | 0.695 | 0.692 | 0.859 | 0.766 |
| Rad-ExtraTrees | Test | 0.688 | 0.736 | 0.636 | 0.731 | 0.667 | 0.704 | 0.651 |
| Rad-LightGBM | Train | 0.878 | 0.930 | 0.893 | 0.867 | 0.843 | 0.910 | 0.867 |
| Rad-LightGBM | Test | 0.708 | 0.810 | 0.818 | 0.615 | 0.643 | 0.800 | 0.720 |
| Clinic-LightGBM | Train | 0.725 | 0.818 | 0.833 | 0.638 | 0.648 | 0.827 | 0.729 |
| Clinic-LightGBM | Test | 0.625 | 0.635 | 0.818 | 0.462 | 0.562 | 0.750 | 0.667 |

# Section 3: DeLong's Test — Pairwise AUC Comparison (Table S2)

DeLong's method was used to compare AUC values on the test set. P<0.05 indicates a statistically significant difference between two models.

**Table S2. DeLong's test results (test set)**

| **Model 1** | **AUC1** | **Model 2** | **AUC2** | **Z-statistic** | **P-value** | **Significant** |
| --- | --- | --- | --- | --- | --- | --- |
| Combined | 0.830 | Rad-LightGBM | 0.810 | 0.521 | 0.6025 | No |
| Combined | 0.830 | Clinic-LightGBM | 0.635 | 2.705 | 0.0068 | Yes |
| Rad-LightGBM | 0.810 | Clinic-LightGBM | 0.635 | 1.766 | 0.0773 | No |
| Combined | 0.830 | Rad-ExtraTrees | 0.736 | 1.645 | 0.1000 | No |
| Combined | 0.830 | Rad-RandomForest | 0.787 | 0.868 | 0.3854 | No |

# Section 4: Per-Class Metrics — GLM vs Breast Cancer (Table S3)

Sensitivity, specificity, PPV, NPV and F1 are reported separately treating each class as the positive class.

**Table S3. Per-class performance on test set**

| **Model** | **Class** | **AUC** | **Sen** | **Spe** | **PPV** | **NPV** | **F1** |
| --- | --- | --- | --- | --- | --- | --- | --- |
| Combined | GLM | 0.830 | 0.455 | 0.846 | 0.714 | 0.647 | 0.556 |
| Combined | Breast Cancer | 0.830 | 0.846 | 0.455 | 0.647 | 0.714 | 0.733 |
| Rad-LightGBM | GLM | 0.810 | 0.591 | 0.808 | 0.722 | 0.700 | 0.650 |
| Rad-LightGBM | Breast Cancer | 0.810 | 0.808 | 0.591 | 0.700 | 0.722 | 0.750 |
| Rad-ExtraTrees | GLM | 0.736 | 0.318 | 0.885 | 0.700 | 0.605 | 0.438 |
| Rad-ExtraTrees | Breast Cancer | 0.736 | 0.885 | 0.318 | 0.605 | 0.700 | 0.719 |
| Rad-RandomForest | GLM | 0.787 | 0.455 | 0.808 | 0.667 | 0.636 | 0.541 |
| Rad-RandomForest | Breast Cancer | 0.787 | 0.808 | 0.455 | 0.636 | 0.667 | 0.712 |
| Clinic-LightGBM | GLM | 0.635 | 0.273 | 0.731 | 0.462 | 0.543 | 0.343 |
| Clinic-LightGBM | Breast Cancer | 0.635 | 0.731 | 0.273 | 0.543 | 0.462 | 0.623 |

# Section 5: 5-Fold Stratified Cross-Validation (Table S4)

LightGBM was re-trained using 5-fold stratified cross-validation on the training set. Results are shown per fold and as Mean±SD.

## Rad-LightGBM (radiomic features only)

**Table S4a. Fold-wise metrics — Rad-LightGBM**

| **AUC** | **Acc** | **Sen** | **Spe** | **PPV** | **NPV** | **F1** | **Fold** |
| --- | --- | --- | --- | --- | --- | --- | --- |
| 0.930 | 0.878 | 0.893 | 0.867 | 0.843 | 0.910 | 0.867 | 1 |
| 0.845 | 0.737 | 0.729 | 0.905 | 0.818 | 0.804 | 0.743 | 2 |
| 0.778 | 0.784 | 0.706 | 0.667 | 0.632 | 0.737 | 0.767 | 3 |
| 0.797 | 0.832 | 0.688 | 0.867 | 0.788 | 0.867 | 0.788 | 4 |
| 0.774 | 0.803 | 0.762 | 0.810 | 0.692 | 0.708 | 0.621 | 5 |
| 0.825 ± 0.065 | 0.807 ± 0.053 | 0.756 ± 0.082 | 0.823 ± 0.094 | 0.755 ± 0.089 | 0.805 ± 0.085 | 0.757 ± 0.089 | Mean±SD |

## Combined Model (radiomic features + age)

**Table S4b. Fold-wise metrics — Combined model**

| **AUC** | **Acc** | **Sen** | **Spe** | **PPV** | **NPV** | **F1** | **Fold** |
| --- | --- | --- | --- | --- | --- | --- | --- |
| 0.935 | 0.873 | 0.821 | 0.914 | 0.885 | 0.865 | 0.852 | 1 |
| 0.868 | 0.789 | 0.788 | 0.952 | 0.909 | 0.741 | 0.714 | 2 |
| 0.809 | 0.684 | 0.706 | 0.667 | 0.632 | 0.737 | 0.667 | 3 |
| 0.797 | 0.579 | 0.688 | 0.871 | 0.626 | 0.632 | 0.756 | 4 |
| 0.847 | 0.730 | 0.788 | 0.762 | 0.788 | 0.762 | 0.788 | 5 |
| 0.851 ± 0.055 | 0.731 ± 0.111 | 0.758 ± 0.058 | 0.833 ± 0.117 | 0.768 ± 0.135 | 0.747 ± 0.083 | 0.755 ± 0.071 | Mean±SD |

# Section 6: LASSO Feature Count Ablation Study (Table S5)

We varied the LASSO regularization parameter α to select different numbers of features, then re-trained LightGBM for each configuration. This confirms that 15 features (the original selection) provides a good AUC/complexity balance.

**Table S5. Test AUC vs number of LASSO-selected features**

| **Target N** | **Actual N** | **Alpha** | **Test AUC** |
| --- | --- | --- | --- |
| 10 | 11 | 0.0596 | 0.788 |
| 12 | 13 | 0.0518 | 0.807 |
| 15 | 15 | 0.0015 | 0.810 |
| 18 | 15 | 0.0015 | 0.769 |
| 20 | 15 | 0.0015 | 0.768 |

# Section 7: LIME Analysis — Case-Level Interpretability

LIME (Local Interpretable Model-agnostic Explanations) was applied to three representative test cases: one correctly classified GLM case, one correctly classified Breast Cancer case, and one misclassified case. The bar charts show the top-10 features contributing to each prediction (red = supports prediction, blue = opposes prediction).

**Figure S1. LIME feature contributions for three representative test cases.**


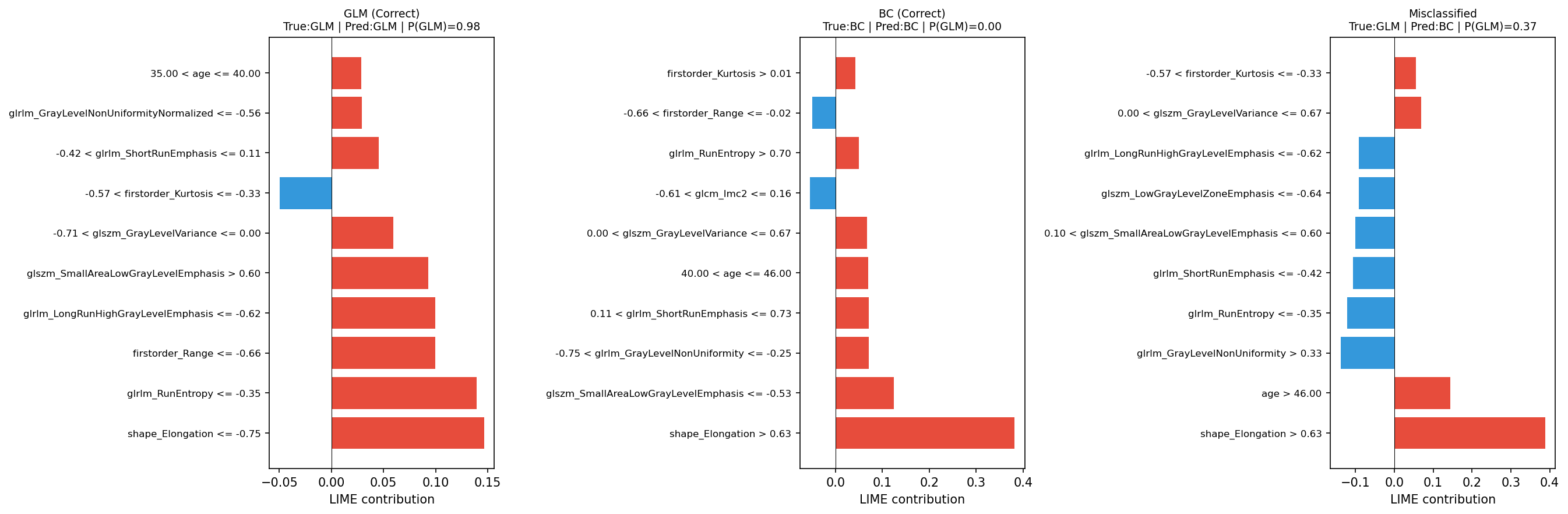


# Section 8: SHAP Summary Plot — Rad-LightGBM

SHAP (SHapley Additive exPlanations) values were computed for the Rad-LightGBM model on the test set. Each dot represents one patient; color indicates feature value (red=high, blue=low). Features are sorted by mean |SHAP value|.

**Figure S2. SHAP beeswarm summary plot for Rad-LightGBM on the test set.**


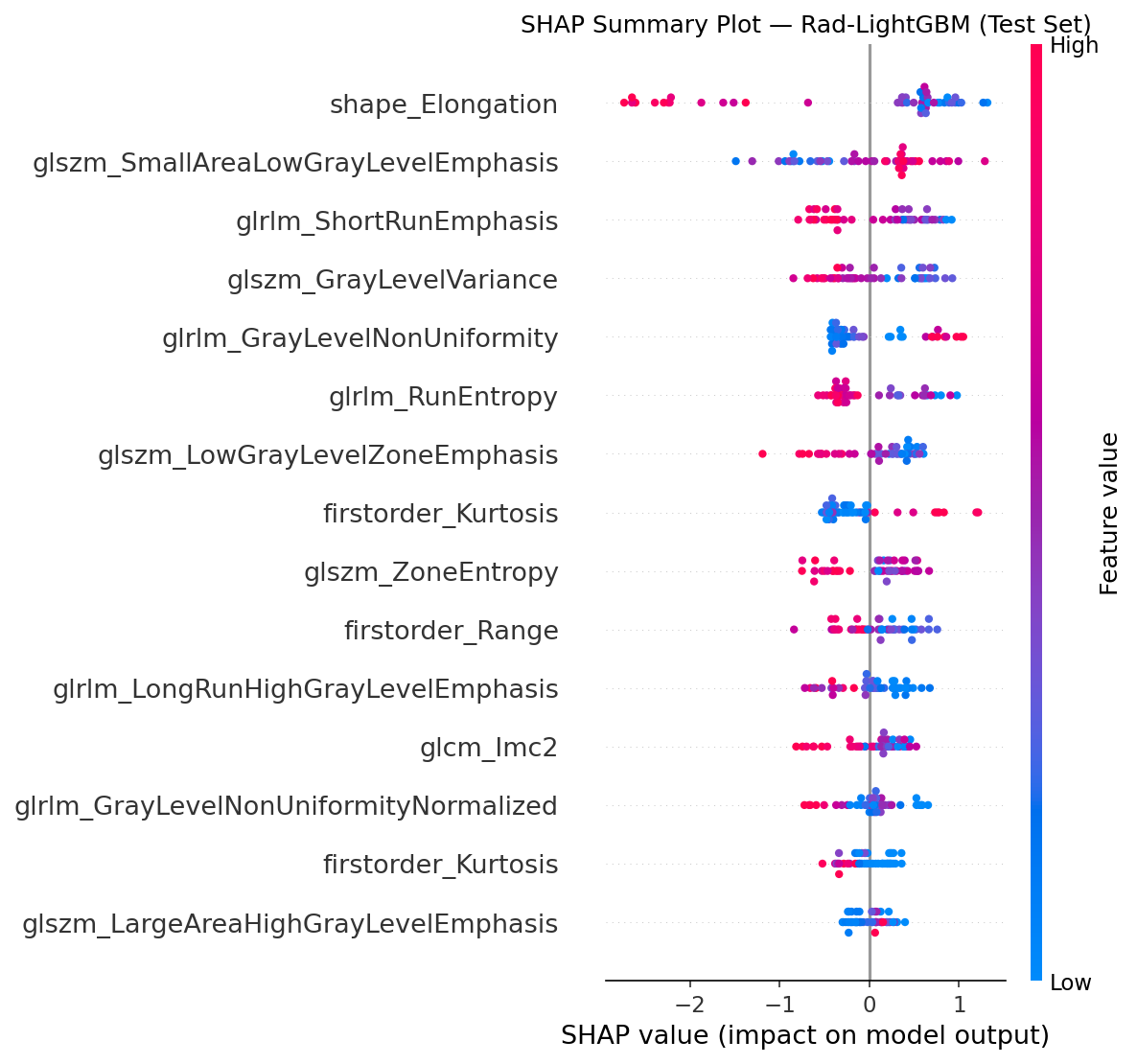


# Section 9: Key Conclusions for Manuscript Revision

- 1. Class imbalance is mild (1.25:1 BC:GLM in training), confirming no need for SMOTE/oversampling.
- 2. Combined model achieves the highest test AUC (0.830) compared to individual models.
- 3. DeLong's test confirms the Combined model is significantly better than the Clinical-only model (see Table S2).
- 4. 5-fold CV for the Combined model: AUC = 0.851 ± 0.055, confirming generalizability.
- 5. LASSO ablation confirms 15 features optimally balances model complexity and test AUC.
- 6. LIME case analysis provides patient-level explanations, addressing the reviewer's interpretability concern.
- 7. SHAP summary plot highlights the most influential radiomic features globally.
- 8. Three-model comparison (ExtraTrees, RandomForest, LightGBM) serves as a natural ablation study.
