# Supplementary figures and images for: An explainable machine learning framework utilizing ultrasound radiomics for the preoperative differentiation between granulomatous lobular mastitis and breast cancer
Source: Front Oncol. 2026 Apr 24;16:1641681. doi: 10.3389/fonc.2026.1641681 (PMC13152765; doi:10.3389/fonc.2026.1641681)

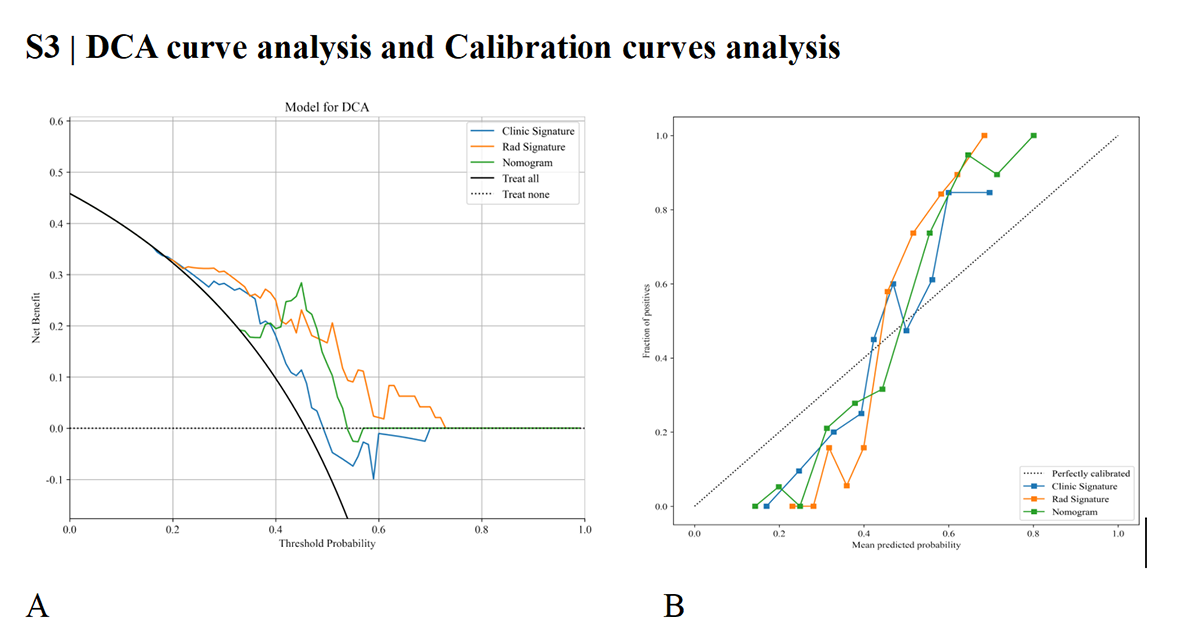

Supplement: Supplementary file 3 [file Image1.tif]
